# Dr. Baillie's Posthumous Works

**Published:** 1826-04

**Authors:** 


*Lectures and Observations on Medicine.* By the late MATTHEW BAILLIE, M.D. Royal Octavo, pp, 242. London, 1825.

ONLY 150 copies of this posthumous work were printed, and that for distribution among the author's particular friends. Although Dr. Baillie's modesty laid an injunction against the *publication* of these papers in the usual acceptation of the word, yet we shall not be deemed sacrilegious in giving them a currency through the medium of our Journal—nor even in commenting upon certain parts of them, in that spirit of liberality by which we should have been guided, had the amiable and able author been alive at the time of our writing.

The present volume consists of an introductory lecture to the course of anatomy delivered in Great Windmill-street—the Gulstonian lectures read before the Royal College of Physicians in the year 1794 on the nervous system—"and some brief observations drawn from the author's own experience upon a considerable number of diseases." It is with these last, of course, that we shall have to do in this place, the lectures being, as lectures usually are, compilations of what is already well known on each particular subject.

It is curious, and certainly unfortunate, that Dr. Baillie only began to record the results of his experience so late as the year 1819, having then been in practice more than 30 years, and having, of course, been witness to innumerable facts and phenomena that would have afforded most valuable data on which to ground principles of practice. All these facts have been lost, or nearly so; for it is evident that these "results of his experience" are rather *opinions* or conclusions, drawn from memory, than the record of *facts* themselves. The *former* change and vary with the observers—the *latter* would have been immutable and imperishable! It is also evident that Dr. Baillie had much less time to record these results than he calculated on when he commenced the practice of committing his thoughts to writing. It was, indeed, a vain hope to expect much leisure after arriving at his eminence, and while he continued at all to practise his profession. When a physician has attained the summit of celebrity, the more he attempts to restrict his practice, the more eagerly will he be sought after, as was clearly exemplified in the instance before us. Dr. Baillie would only meet in consultation. What then? The calls for consultation were as numerous as the hours, or rather the half-hours of the day!

It was not his lot to have

"A youth of labour, and an age of ease."

And how is it, we might ask, that, of all classes of society, the physician alone, seldom or never retires "to husband out life's taper at its close," but, on the contrary, lingers to the last in the chambers of sickness, sorrow, and death? Is it that this last grim messenger of fate has appeared in so many forms that his presence no longer excites a sensation or reflexion in the physician's mind? No. The physician is not a whit less loth to "leave the warm precincts of the cheerful day" than any of his patients. Is it that the love of lucre grows with his growth, and that his palm, so often graced with the image of his *sovereign*, still itches for the Royal touch? Not so. There is less avarice and more generosity among physicians generally, than among the members of the other two learned professions. Is he afraid of *ennui* if he quits his daily rounds before the night of death closes on his labours? No. The physician has as many resources in retirement as the lawyer, the divine, or those of any other rank or class of society. What is it then, it may be asked, which binds the physician to a calling so laborious and apparently disagreeable, after realizing a fortune which he can never expect to spend? There are several reasons for this apparent anomaly. In the first place, the force of *habit* is not less operative on the physician than on others of his species—for how often do we see individuals cling to the senate, the bar, and the pulpit, long after their best friends and their own reputations demand a retreat. But there is something peculiar in the case of the physician. In the eyes of the community at large, length of years is only supposed to produce accumulation of knowledge, and he is sought after till the decay of *physical* strength in himself, and of confidence among his *professional* brethren, forces him at last, reluctant and lingering, from the stage of public life!

But there is yet another reason why the physician (and by the physician we mean the medical man generally,) clings to practice longer than those of other professions. It has not been noticed, as far as we recollect, and yet it is the most powerful motive of all. This is VANITY. The practice of medicine gives a man a much greater degree of private influence, and a much wider range of individual acquaintance, contracted too when all the sympathies and emotions of the heart are in full play, than any other profession whatever. The statesman is known in the senate—the lawyer at the bar—the divine in his pulpit—but the physician is known in the chamber of affliction, where the whole history of the sick man's life, as well as many important secrets of the family are confided to his breast. Are not these circumstances well calculated to confer a degree of importance on the physician, not only in his own eyes, but in those of his patients, far beyond what his rank in life would naturally produce? Such are the ties which bind him strongly to life—to that kind of life in which his vanity and self-love are gratified to the latest period of his career. No wonder, then, that he quits the feverish drama of human existence late and reluctantly. Vanity, like hope, springs eternal in the breast of man as well as of woman—and, indeed, we have doubts whether it does not acquire strength in proportion as the other passions subside and vanish.

For this digression we have to apologise, and shall now proceed to business.

1. *Apoplexy*. After some brief observations on *head-aches*, in which Dr. Baillie avers that he has seldom seen much good, and often harm, from repeated local or general bleedings, and recommending temperance in regimen, bitters, and gentle aperients, with the application of cold to the head, our author comes to the subject of apoplexy. Dr. B. considers the *severe forms* of this disease as owing to actual effusion of blood, generally into the medullary substance of the brain, near one of the ventricles—while the *milder* forms are dependent on *distention* of the cerebral vessels by their own contents. He has seen only one instance of fatal apoplexy, where there was no extravasation, and only vascular distention. We confess that this observation surprises us a good deal, and leads us to suspect that Dr. Baillie did not rigidly prosecute pathological investigations after he had become immersed in extensive practice. We have seen three or four cases ourselves where men died, and that very quickly too, with all the symptoms of severe apoplexy, and where no rupture, but strong turgescence only could be detected after death. Numerous cases of this kind are on record, both by the older and the more recent writers.

To the following therapeutical remarks we can have no objection.

"The chief remedy in apoplexy is large bleeding, to be repeated according to circumstances. Topical bleeding by cupping and leeches is likewise often of use. The next remedies in importance are purgative medicines of considerable power, and acrid glysters. The head should be kept high or elevated, and cold may be applied with advantage to the top of the head. If the patient should recover by these means, the best plan of management, in order to escape from another attack, is to live almost entirely throughout future life upon vegetable food, and to abstain from wine, spirits, and malt liquor. It will be of considerable advantage to avoid any strong or long-continued exertion of the mind. In a few instances, when the full state of the vessels of the brain had for some time subsided, I have derived considerable advantage from the moderate use of tonic medicines, and more especially of steel." 168.

2. *Hydrocephalus.* Our author has known, in his own experience, of but one instance of this disease cured, when fully formed.

"In this case all the symptoms were well marked, and the disease had made such progress that squinting and an irrégular pulse had taken place. There had been no peculiar treatment, except that mercurial ointment was applied daily to a considerable sore on the upper part of the head, which had been produced there by a blister. The individual is now alive, and is a young lady of good talents, which she has highly cultivated.

"I have seen a few cases, in which there appeared to be a strong threatening of Hydrocephalus, that got well by the application of leeches and blisters to the head, and brisk mercurial purges; but I cannot determine whether these cases, if less actively treated, would have terminated in true Hydrocephalus or not." 169.

3. *Epilepsy.* Dr. Baillie believed that this disease had become much more frequent within the last twenty years than formerly. If so, he thought it might be accounted for by the progress of luxury, which must render the nervous system more irritable. He has known of very few instances of epilepsy radically cured; but a considerable number in which the intervals had become much longer. The medicines which he found most beneficial, were the argentum nitratum, the viscus quercinus, and the oleum succini. The first is the most powerful; "but when used a good many months, it tinges the skin of some individuals of a dark colour." He saw two instances of this in his own practice. Temperance is recommended in this disease, with open bowels, and cold to the head. We believe that no instance of cutaneous discoloration has taken place unless the nitrate of silver was continued longer than three months. In several instances of epilepsy, apparently unconnected with any visceral, or other local disease, we have given the tincture of lytta, to such an extent as to produce some heat in making water, as an important adjuvant to the nitrate of silver.

4. *Tic Douloureux.* This, in Dr. Baillie's experience, is a disease on the advance in our own times, as are most of those of the nervous system. Dr. B. never knew the disease completely cured, though temporary respite was afforded by division of the nerve, by bark, and by arsenic. Dr. Baillie did not know, at the time of writing, that in *iron*, we should find a better remedy than in *steel*, for tic douloureux.

5. *Lymphatic Glands of the Neck.* In swellings of these glands our author found good effects from sarsaparilla and soda, with some form of steel—but more powerful effects from sea-air and sea-bathing—and most of all from the air and waters of Malvern. Of bronchocele Dr. Baillie had not much experience, and did not know the efficacy of iodine in this disease.

6. *Chronic Laryngitis and Tracheitis.* This often produces phthisis. Medicines have but a very gradual influence upon the complaint, and sometimes none at all. Repeated leeching and blistering are useful.

"But perhaps the most useful remedy is a small seton inserted under the skin of the side of the neck, very near the larynx. Internal medicines often produce very little good effect; but the medicine which I have found upon the whole to be the most beneficial has been the extractum conii. I have sometimes directed five grains of it to be taken three times a day for many weeks together, with manifest advantage." 174.

7. *Quinsy.* We have repeatedly verified the following remark on this apparently trifling, but yet very distressing malady.

"I have but one observation to make with regard to this disease, which is of some little importance. It is usual to endeavour throughout the course of it to prevent suppuration from taking place, by the repeated application of leeches under the angles of the lower jaw. It is certainly very desirable that suppuration should be prevented, and that inflammation of the tonsils should gradually subside by resolution. I have found, however, by experience, that suppuration is by such means very often not prevented, but only that inflammation proceeds more slowly to this issue. Hence the patient suffers for a considerably longer time; and the suffering in this disease is often very great. If, therefore, one or two applications of leeches do not lessen materially the inflammation of the tonsils and velum pendulum palati, I should recommend the progress of the inflammation to be encouraged by the inhaling of warm vapour into the mouth, and the application of poultices to the external fauces. In this way the disease will go through its progress more quickly, and the patient will suffer much less." 175.

9. *Phthisis.* In the course of a long experience Dr. Baillie "has known one or two cases of patients who recovered from phthisis which was apparently fully formed." But even with regard to these cases, our amiable and candid author thinks it probable that he may have been mistaken! This is a melancholy prospect for the phthisical patient, and holds out few hopes of the "sanability of consumption." Dr. B. has known a good many instances, however, in which persons threatened with consumption, have recovered by going into mild climates, or even into Devonshire or Cornwall, but in no instance where the disease was decidedly formed. We are happy to say that we have seen one case of as unequivocal phthisis as we ever before witnessed, completely cured by one year's residence in Penzance. We attended the patient for some weeks before she went there, and have repeatedly seen her since. We do not think, therefore, that we have been deceived. We have heard of many others who have recovered, but never before saw such a decided instance as this.

In respect to the numerous cases of recovery which we read of, under particular modes of treatment, we have little confidence in the narratives. Most of them are deceptions or misrepresentations—the *former* arising from mistaking affections of the mucous membrane of the lungs for tubercular excavations—the *latter*, from motives which we dare not permit ourselves to characterise by their proper designations.

"When no active inflammation is going on in the chest in phthisis, I have sometimes found advantage from patients being allowed to take a little white fish or light animal food at dinner. In a very few instances I have found benefit derived from taking one, or even two glasses of wine diluted with water, after dinner; but wine is generally improper.

"I have known of no medicine which has been of permanent and substantial use in phthisis; but I have sometimes found a good deal of temporary advantage derived from myrrh, from ammonia, and from light bitters united to the acetic acid. The frequent repetition of blisters, or a seton inserted under the skin in some part of the chest, are occasionally of considerable use." 179.

10. *Hydrothorax.* Dr. Baillie found this dropsy more amenable to medicine (when not dependent on diseased structure of the heart or lungs) than either ascites or ovarian dropsy. But, unfortunately, the instances of simple uncomplicated hydrothorax are very few indeed. The medicine which he found most beneficial was mercury combined with squills and digitalis. In many instances this combination mitigated, or, for a time, removed the disease. "There has been some advantage from the mercury affecting slightly the salivary glands. Squills and digitalis are by themselves much less efficacious than when combined with mercury." 180. Dr. Baillie does not recollect one instance of hydrothorax being permanently cured. This can only have applied to those cases which were complicated with or dependent on, an organic disease of the heart or lungs. There are certainly instances of acute dropsy of the chest cured —we mean of hydropic effusion from inflammation of the serous membranes. But this is a piece of pathology little attended to by Dr. Baillie or the modern physicians of the old school.

11. *Palpitations of the Heart.* There are few phenomena which puzzle, perplex, and lead into error the inexperienced (and sometimes the experienced) practitioner, so much as inordinate action of the heart. He sees (or thinks he sees, like the school-boy in the church-yard) some terrible cause for this tumult in the central organ of the circulation, and frames his portentous diagnosis and prognosis accordingly. In the pride of his penetration he renders miserable, for a time, the friends —and, by his direful countenance, damps the spirits of his patient. But ultimate recovery not seldom *disappoints* his fears —and the physician is *mortified* at his own success! This may seem hard language, but we appeal to the *heart* itself for its truth.—Hear we then what Dr. Baillie has to say on palpitations.

"Palpitation of the heart may take place at any period of life; but it is more common at an early period than any other, as for instance from fifteen to twenty-five years of age. Perhaps, too, it may be more common in females than in males, but of this I am not very certain. At an early period of life it does not in general depend upon any diseased structure of the heart, but either on a morbid irritability of the nerves of this organ, or upon some imperfect state of digestion. When it takes place from either of these causes, it always continues for a long time, (often, more or less, for two or three years), but at length generally subsides. Rest of body and quietness of mind are two of the chief means which contribute to remove this disease. All quick motion of the body, and more especially walking up ascents, increases the complaint, and should as much as possible be avoided. Every thing which tends to excite or harass the mind has the same effect, and should be shunned whenever it is possible. To rest of body and mind should be joined very temperate diet; and when this general plan of management has been continued for many months, or perhaps for a year or two, the disease usually subsides. Digitalis has sometimes been useful in mitigating this complaint, but frequently it produces no good effect.

"Where the palpitation depends either altogether or chiefly upon the state of the stomach, it is gradually removed by temperance, by improving the condition of the stomach, and by keeping the bowels free from costiveness. I remember one case in which palpitation of the heart had taken place, and had continued for six months, in consequence of gout having attacked this organ. In this case the palpitation ceased suddenly and entirely when the gout attacked one of the feet in a full and decided form. This person is now alive, and has continued generally in good health, although it be nearly twenty years since the attack of palpitation.

"In some young persons palpitation depends upon an enlargement of the several cavities of the heart, produced not unfrequently by rheumatism attacking this organ. This cause of enlargement of the heart was overlooked by the physicians of this country, till it was discovered by the sagacity of my esteemed friend the late Dr. David Pitcairn. The enlargement in general goes on increasing till life is destroyed; but I have known two cases where the enlargement stopped at a certain point, the increased action of the heart in a great measure subsided, and the patients acquired a tolerable share of health. They are both now alive, and they have the prospect of living, with care, to the ordinary term of life. Such a fortunate issue is very rare; but the disease may be generally retarded in its progress by much rest of body, quietness of mind, and a very temperate mode of living. Wine and every other fermented liquor should be avoided, and patients under such circumstances should live almost entirely upon vegetable food.

"At the middle and more advanced periods of life, palpitation of the heart often depends upon a diseased structure of some of its valves. This condition of the heart does not admit of any remedy, but must gradually become worse, until life be extinguished. But the symptoms may be mitigated, and the progress of the disease retarded by little exertion of the body, by great temperance, and by a few ounces of blood being occasionally taken from the arm." 185.

In respect to angina pectoris, our author believed that it almost constantly depends upon ossification of the coronary arteries of the heart. This was a great oversight—and tends, among many other circumstances, to convince us that Dr. B.

paid little attention to pathology, when he was in that station that gave him the opportunity of comparing the *post mortem* appearances with the previous living phenomena—the only sure basis for the study of this important branch of our science.

"I have met with two cases," says Dr. B. "however, in the course of my medical experience, in which symptoms exactly resembling those of angina pectoris depended upon an imperfect digestion; and the patients ultimately recovered entirely, by correcting the disordered condition of the stomach." 185.

We have seen several cases where all the symptoms of angina pectoris were exquisitely marked, and where the organic lesions discovered after death were very different from coronary ossification. One remarkable instance of this kind we shall shortly lay before our readers.

12. *Ascites.* This disease, whether dependent on organic affections of the abdominal viscera or not, Dr. B. has seldom known to be cured. The ordinary diuretics, in his experience, had little effect upon it. The most influential medicines, in his opinion, are supertartrate of potash, and small doses of elaterium. In two or three instances the dropsy disappeared spontaneously, when all medicines failed.

Of venesection in ascites, Dr. B. had no experience, but does not doubt its utility in some cases.

13. *Peritonitis.* When this disease was not connected with epidemic influences, Dr. B. found it to give way to bleeding and purging like other inflammations—but more to local than to general depletion. Calomel and the neutral salts were the purgatives most employed by our author.

14. *Some Affections of the Stomach.*

"There is no complaint more common in this country than an imperfect condition of the functions of the stomach. This generally shows itself by more or less of flatulence, by acidity, by a bitter taste occasionally felt in the mouth, and often by some degree of costiveness. This condition of the stomach generally arises from something wrong in the quantity or quality of the food, from anxiety of mind, and from a due degree of exercise not being regularly taken. It makes its progress very gradually, continues always for some months, and often even, more or less, for years.

"The first object of attention should be to remove as far as possible the causes which produce it. Every kind of food should be avoided which the patients may have found, from their own experience, to have disagreed with their stomach. Most commonly animal food that is very fat, or much salted or fried, is difficult of digestion, and should either be eaten very sparingly, or should be altogether avoided. Young and white animal food is in general more difficult of digestion than what is brown and of middle age. The vegetables which are eaten should be very well boiled, and should be taken sparingly by such persons as are subject to flatulence or acidity. The waxy potatoe is almost constantly very difficult of digestion, and in general should be avoided altogether. There should never be so much food taken at a time as to give the feeling of fullness or distention in the stomach; and, except under very particular circumstances, there is no advantage in eating oftener than three or four times in twenty-four hours. The best common beverage in disordered conditions of the stomach is water, or toast and water; and three or four glasses of wine may be taken at or after dinner, according to the habits of the patient, or other circumstances. That wine is to be preferred which agrees best with the stomach, of which he is himself the most competent judge. Daily exercise is almost constantly necessary in order to preserve good digestion. Riding on horseback is upon the whole the best, for it gives a motion to the abdominal viscera, which no other exercise is capable of; but walking is also very useful. A combination of the two is preferable to either; for riding on horseback chiefly exercises the abdominal viscera, and walking chiefly exercises the limbs and the thoracic viscera. Anxiety of mind should be avoided, whenever it can fairly be done; but it is often impossible to take advantage of this remedy.

"With respect to medicines, there are none for this complaint which can be called specific. The most beneficial, however, which I have known are rhubarb, and some form of bitter medicine combined with alkalies. Eight grains of rhubarb formed into pills with soap, taken every night at bed-time, and some bitter,—as infusion of cascarilla, calumba, quassia, or gentian, with some grains of soda or potassa dissolved in it, taken in the morning and before dinner,—will often be very useful in this kind of disordered stomach. These remedies should be continued for five or six weeks at a time, should be omitted for two or three weeks, and occasionally resumed. If the alvine evacuations should be considerably lighter in their colour, or much darker than natural, mercury, given in moderate doses, and not for so long a time as to injure the constitution, will often be of great use. The large and indiscriminate employment of mercury in complaints of the stomach has, I think, been often very hurtful. Where acidity has been particularly prevalent in the stomach, I have sometimes found it more effectually corrected by the diluted mineral acids than by alkalies. Ten or twelve drops of the diluted sulphuric or diluted nitric acid, mixed with an infusion of some bitter, and taken twice a day, will sometimes be very beneficial in this condition of the stomach.

"There is an affection of the stomach in which the digestion is very imperfect, and in which considerable quantities of a transparent viscid mucus is formed. This often produces nausea, and is occasionally brought up by vomiting. According to my experience, this condition of the stomach has been frequently little benefited by medicine; but sometimes I have found the *tinctura benzöes composita* of considerable use. A drachm of it may be taken mixed with water and some mucilage of gum acacia, three times a day.

"There is another affection of the stomach less common than the former, but far more serious, viz. where the stomach throws up in large quantity a fluid like cocoa. A quart of this fluid will often be thrown up at a time, and this will frequently be repeated for many days together. This condition of the stomach is sometimes connected with a diseased state of the liver, but sometimes it is independent of it, there being, at least apparently, no disease in this latter organ. In several instances it has proved fatal; but in others, and especially in two cases which I recollect, the complaint subsided for several months at a time, and the persons enjoyed in the interval tolerable health. This state continued many years, and the patients are still alive. In one case I had an opportunity of examining the condition of the stomach after death. It was very capacious, and was half filled with this brown fluid, but did not appear to be at all diseased in its structure. The neighbouring viscera, as the liver and spleen, were, as far as I recollect, perfectly sound. The fluid would appear to be formed by a diseased secretion of the inner membrane of the stomach, without any apparent morbid structure.

"This disease, according to my experience, is but very little influenced by medicine or by diet. In two or three cases some benefit seemed to be derived from astringent medicines combined with moderate doses of opium,—as, for instance, from tincture of kino, or tincture of catechu, with a few drops of laudanum, taken three or four times a day. The bowels should be at the same time kept free from costiveness.

"In some cases the stomach will lose almost entirely the power of digestion; the patients will become pale and emaciated, and appear as if they were affected by some fatal visceral disease; at the same time no morbid structure in the region of the stomach or liver can be detected by the most attentive examination. In some of these cases the patients have been completely restored to health by a course of the Bath waters."* 194.

---

* It is in this last class of affections that the sulphate of quinine, in small doses, becomes a most powerful restorer of gastric action and chylification. The following is a formula, which we find of almost universal application. R. Tinct. Gentianæ Comp. ℥iss.

| | |
|---|---|
| ——— Cinchonæ Comp. | ℥ss. |
| ——— Capsici, | ʒi. |
| Sulphatis Quininæ | gr. viij. |
| Acidi Sulph. Aromat. | gtt. vj. |

Misce, capiat Coch. Minut. i. vel ii. ter die ex aqua vel aqua Hordiata. Or a solution of the above kind, entitled "Solutio vel Tinctura Quininæ," may be kept by the general practitioner, and from one to two drachms given in a draught of infusion of cascarilla or ginger, thrice a day, with two or three grains of the pilula hydrargyri with or without aloes at night, so as to keep the bowels soluble. The effects of this remedy in dyspeptic complaints are truly surprising.—*Rev.*

On inflammation of the bowels and on dysentery, Dr. Baillie makes some brief observations, but none that require notice. The therapeutical directions are feeble and inefficient.

15. *Biliary derangements.* Dr. Baillie's observations on this extensive class of disorders are rather meagre, and common-place—an observation which certainly applies to almost all the papers in this volume, and for a reason which is sufficiently obvious. The following passage is the only one which we shall extract, as a rather favourable specimen.

"There is sometimes a greater fullness and greater sense of resistance over the whole region of the liver than natural, with more or less of tenderness upon pressure. This arises from some chronic inflammation of the substance of the liver. In such a case, the repeated application of leeches to the seat of the liver, and the occasional application of a blister, are often of the greatest use. A mild course of mercury should be recommended, so as in some degree to affect the constitution; and this should be administered both externally and internally. It should not, however, be carried beyond the necessity. Long and repeated salivations will seldom be required, and often have done much and permanent injury to the constitution. When the liver has become soft, has lost its tenderness and resumed its natural size, the mercury may be given up. If the liver shall not have returned altogether to its natural state, and the constitution appears to be suffering from the course of mercury, a seton may be inserted under the skin in the region of the liver, and the mercury may be given up or suspended. In some cases I have found a fullness of the liver which had eluded the effect of mercury, to be removed by a seton. The administration of purgatives is of great advantage in all such cases, and the Cheltenham waters are often highly beneficial." 200.

Under the head of "abscess in the liver," Dr. B. makes some observations which, we confess, have rather surprised us. We agree with our amiable author, that hepatic abscesses will often do well when they take an external direction; also, that it is possible a patient may recover after an abscess has found its way through the diaphragm into the lungs, whence the matter is expelled by expectoration. Unfortunately, however, recovery is very rare under such circumstances, as we have not known above three or four instances of the kind among a considerable number. In respect to the following remark, we confess ourselves extremely sceptical.

"When the abscess communicates with the stomach, the matter is sometimes discharged by vomiting, and sometimes by the bowels. In this case, too, the patient will not unfrequently recover; and the same observation may be extended to an abscess of the liver which communicates both with the stomach and the lungs, although the circumstances are more unfavourable in this than in the other two cases." 201.

We verily believe that no one ever recovered where an hepatic abscess communicated with both stomach and lungs. In fact, we have never seen an instance of recovery where the matter burst into the stomach.

16. *Gall-stones.* Dr. Baillie properly remarks, that we have no power either of dissolving biliary concretions or facilitating their expulsion from the ducts of the liver. If inflammation take place we can control it—and the exquisite pain produced by the presence of these bodies in the ducts can be mitigated by large doses of opium. Our author takes no notice of the *prevention* of the disease, though this is of the greatest consequence, since we have so little control over it when actually formed. We conceive that derangement of function in the biliary organ is the cause of biliary calculi, and that attention to this derangement will often prevent their formation. In several people who were subject to repeated attacks of gall-stones, the disposition has been entirely got rid of by improving the functions of the liver and digestive organs generally.

Passing over some uninteresting remarks on affections of the spleen, pancreas, and kidneys, we shall stop for a moment on the subject of

17. *Diabetes.*

"I have in the course of my medical life seen a good many instances of this formidable disease. Of late years a considerable proportion of such cases have got well under my care, or have had the symptoms very much mitigated. The most successful plan of treatment has been, to give considerable doses of opium combined with rhubarb or some other bitter: fifty drops of laudanum, for instance, may be given three or four times a day, mixed with some infusion of rhubarb or infusion of calumba. The rhubarb may also be given separately in the form of pills. Under this treatment the disease will often gradually subside, and at length cease altogether. It is, however, very apt to recur, and therefore this plan of treatment, in more moderate doses, should be continued for some months after the patient is apparently well. Bleeding from the system generally, and topical bleeding from the loins, are often useful; for the blood-vessels of the kidneys in this disease are generally more or less distended with blood. The diet should be temperate, and should consist chiefly of animal food; and the best kind of drink is, upon the whole, Bristol water." 220.

18. *Affections of the Bladder.* Dr. Baillie adverts to the temporary paralysis of the bladder which we occasionally observe in young females who too long retain the urine, from shame or necessity. We lately saw an instance of this inability to make water, not from paralysis of the bladder but from spasm of the sphincter of that organ. The young lady, who was of a very nervous temperament, was subject to severe cramps or spasms of various muscles of the extremities and trunk. At these times the sphincter of the bladder would be thrown into such violent contraction as to set the expulsive powers of that organ completely at defiance. The greatest difficulty was experienced in introducing the catheter, but the moment it entered the bladder, the water gushed forth with great force, shewing that the muscular fibres of the viscus had lost none of their power. The water, at one time, required to be drawn off twice a day for a fortnight—the balance between the sphincter and the bladder then returned, and all went on well again.

19. *Fevers.* Dr. Baillie offers but a few observations on the subject of fever. He considers that the most successful method of treating fevers which aims at removing or mitigating the symptoms as they arise. And truly so it is. We cannot stop a fever when once formed—and what better can we do than watch the symptoms, and guard important organs from the effects of the tumult that is going on in the system. This is the whole sum and substance of the secret of treating continued fevers. When they become intermittent, that is another affair. We have then powerful specifics for arresting their course.

We shall extract the following passage as containing good advice, and a candid avowal at the close of a long professional life.

" The most successful method of treating these fevers, as far as I have seen, is to remove or mitigate the symptoms as they arise. The symptoms denoting an affection of the brain should be relieved, as speedily as possible, by cupping, leeches, and the application of cold to the head. Cloths dipped in iced water, and kept almost constantly applied to the shaved scalp, have appeared to me more effectual in removing delirium than any other remedy.

" When there is pain in any part of the chest or difficulty of breathing, these symptoms should be relieved as soon as possible by cupping or leeches, or blisters, and by saline medicines.

" If there be any pain in the abdomen, or any symptoms denoting an affection of the liver, the stomach, and the bowels, these are to be relieved by their appropriate remedies.

" If there be too vigorous a circulation over the body, without any apparent local affection, it may be corrected by a very cautious bleeding from the arm, by purging, and by saline medicines. If the actions of the constitution be feeble, they may be strengthened by tonic and stimulating remedies, the best of which I believe to be wine in suitable doses. By this mode of treatment fevers will often terminate favourably, which otherwise would have been fatal.

"During the greater part of the time in which I have practised medicine, physicians in general, *and myself among that number*, have, I believe, been too sparing in taking away blood in typhous fever. It was hardly ever directed to be taken away from the arm, and not often locally, except by the application of leeches to the head. Of late years many physicians have gone into the opposite extreme, and have taken away blood too profusely. In the course of a few years this remedy, like every other, will find its proper level." 240.

Here end the posthumous writings of our amiable and experienced author. It is evident that they contain nothing novel or in the least above the ordinary level of a common observant practitioner. Their only utility is to shew what were the practice and opinions of a man whose advice was sought by almost every one who was out of health and could spare a guinea for the purchase of a prescription. These writings prove, what indeed is well known, that neither genius nor splendid talents *were* essential to success—superlative success, in medicine. There was no indication of either the one or the other, in the practice or writings of Dr. Baillie. Good sense, correct ethical conduct, and common observation, are the only characteristics of this lamented physician. But these were capable of conducting him to a pinnacle of fame and fortune, which *no future physician will ever attain again in this metropolis*. And why not, it may be asked? The reason, we think, is obvious enough. Thirty or forty years ago more ordinary talents and attainments than those professed by Dr. Baillie, were sufficient to bring a man prominently forward, under such auspices as those of the Hunterian family; but the case is now altered. The diffusion of knowledge among all ranks of the profession, through the instrumentality of the press, will never more permit any individual to gain such an ascendency as Dr. Baillie possessed. There may be an aristocracy—but never, hereafter, a king among physicians. Even the aristocracy is threatened. Democracy is every day gaining ground—that is, a greater degree of equality is daily obtaining not only among the various individuals of the same rank, but even among the different ranks themselves. The thirst of knowledge is becoming universal—the facilities of acquiring it are multiplying—and, consequently, such a competition for reputation will be constantly exerted as must completely exclude *supremacy*. We are not sorry to see this state of things fast approaching. It is an event extremely beneficial to society at large. There is this consolation for talent and industry—that although the possessor can never hope to rise by the space of head and shoulders above his neighbours, yet any degree of elevation beyond the mean level will, in future, be more indicative of superior talent, than was a giant's height in the days of yore, when medical education and science were, comparatively speaking, in the hands of a few.

